# ‘Cucumber sandwiches that repeated’: Loneliness and melancholia in Elizabeth Taylor's *Mrs Palfrey at the Claremont*

**DOI:** 10.1111/criq.12728

**Published:** 2023-06-06

**Authors:** Akshi Singh

**Affiliations:** ^1^ Department of English, School of Critical Studies University of Glasgow Glasgow UK

It remains a mystery to me why Elizabeth Taylor, onetime member of the Communist Party – ‘I did not see why economic freedom would not lead to the other more important liberties – of speech & thought & expression . . . a woman respected first as a person, not as a machine for reproduction’ – is hardly considered a politically engaged novelist.^1^ It really seems like a case where a writer's ability to describe hats has worked against her, as though someone who knows the details of women's clothing, and describes with precision the running of a household, can have little to say about the politics of her time. It is true that, with the exception of her first novel, *At Mrs Lippincote's* (1945), communists or political radicals don't occupy a prominent place in her writing. And even in this book, the depiction of the Communist Party is irreverent, a woman attending a party meeting misquoting Auden to herself to keep going (‘today the expending of powers on the ephemeral pamphlet’), only drawn to attention by horror, ‘Hindus tied to trees by their hands, their toes barely touching the ground, hanging there in the ferocity of the sun, a punishment for – and this was the point – trade union activity.’^2^ But the mostly conservative, sometimes sequestered characters that Taylor creates in her other novels are no less politically interesting than Communist Party members. Not least because of – to use a somewhat old‐fashioned phrase – Taylor's historical consciousness, one that includes, much to her credit, awareness of the distinction between elasticised stockings and those held up by garters.

I read most Elizabeth Taylor's thirteen novels during the COVID‐19 lockdowns in Britain, and the ones I had already read, I reread. Taylor is excellent at plot, by which I mean its concealment – events seem to be just a flow of actions and consequences. Living alone, I was sometimes lonely. I borrowed a sense of movement, of time as something dynamic, from the novels. In those circumstances, one book stood out: *Mrs Palfrey at the Claremont*. This is amongst Taylor's best known novels, and was nominated for the Booker Prize in 1971. Here Taylor makes something of a page‐turner out of the experience of stuck time, offering an intimate portrait of boredom and loneliness. This alone is remarkable, but in this essay I want to examine the ways in which Taylor's novel situates the roots of this loneliness in Britain's loss of empire, a reading of the nation that is all too relevant in the present.

The eponymous Mrs Palfrey is a widow of some means, who has come to live in South Kensington, at the Claremont Hotel: ‘there were spotted laurels in the window‐boxes; clean curtains – a front of emphatic respectability’.^3^ The Claremont is a place, as Mrs Palfrey will soon discover, where other people from her generation and class come to wait out their days. For the residents it does indeed offer ‘a front of emphatic respectability’ – for the disregard of family, the absence of friends, and the looming spectre of an anonymous and expensive nursing home. Time passes slowly at the Claremont, there are never enough activities to fill the empty hours:

As she waited for prunes, Mrs. Palfrey considered the day ahead. The morning was to be filled in quite nicely; but the afternoon and evening made a long stretch. I must not wish my life away, she told herself; but she knew that, as she grew older, she looked at her watch more often, and that it was always earlier than she had thought it would be. When she was young, it had always been later.^4^

Taylor's writing takes us into Mrs Palfrey's experience of time. Already waiting for breakfast, she contemplates a day of waiting around. Observe the punctuation in the passage above, the commas in particular give pause, interrupt the reading. This halting movement through Mrs Palfrey's thoughts, the unwelcome and awkward pockets of enforced quiet between the clauses are much like Mrs Palfrey's day, where each errand is eked out for as long as possible: ‘she made it last as long as she could so that later might seem sooner’.^5^ Frequent mentions of the time accompany descriptions of Mrs Palfrey's first days at the Claremont, the passing of time marked in hours and quarters. John Wiltshire's discussion of Jane Austen's punctuation comes to mind when thinking of Taylor, her ability to ‘lodge emotion’ in these breaks.^6^ Her contemporary and friend, the writer Elizabeth Bowen, had similar felicity with the unsaid, leaving sentences incomplete, particularly in the narration of dialogue. Like Austen and Bowen (‘Soul sisters all’, according to Anne Tyler), Elizabeth Taylor knows what to do with a silence, the potential of a pause, and the suggestive possibilities of the unsaid.

Mrs Palfrey's empty hours intersect with those of the other regular residents of the hotel. In addition to Mrs Palfrey there are three widows and a widower: Mrs Arbuthnot, Burton, and Post, and Mr Oswald. Though it is clear that each person is solitary, there is shame associated with this condition and the residents cannot provide anything but the most attenuated companionship to each other: ‘at the Claremont, days were lived separately’.^7^ In the elaborate concealment of loneliness at the hotel, the having or not having of visitors acquires painful importance. When Mrs Palfrey's grandson doesn't visit she is left exposed to the comments of Mrs Arbuthnot, who ‘condoled with her spitefully’.^8^ Lying awake at night, ‘feeling panic at her loneliness’, she is haunted by the thought of a Miss Benson who once lived at the hotel, never had any visitors, ‘was entirely alone’.^9^

The widows of the Claremont suffer the loss of their husbands, their former status, their abandonment to the mercy of relatives and visitors who ‘come for a while’, and go ‘relievedly away’.^10^ From the tone of Mrs Arbuthnot's voice when she speaks of her late husband, Mrs Palfrey discerns that she ‘blamed him for dying, for leaving her in the lurch’.^11^ Mrs Arbuthnot is without a husband to complain to the hotel's management about the size of her small room, her own protests go unheeded, the hotel ‘stuffed elderly women into the worst bedrooms at a price they could just afford’.^12^ ‘We poor old women have lived too long’, says Mrs Arbuthnot. ‘As one gets older life becomes all take and no give’, remarks Mrs Post on another occasion. ‘Be independent; never give way to melancholy; never touch capital.’ This is Mrs Palfrey's motto, and she is engaged in a purposeful battle to uphold it, but she too is filled with regret for her former life: ‘there was no husband to take her arm across a road, or to protect her from indignity when she failed’.^13^

South Kensington is an excellent location for literary loneliness, and *Mrs Palfrey at the Claremont* can be seen as part of a tradition of fiction set in West London that is concerned with relations between women, the knife twisting concealed within polite conversation, and the exploration of feminine solitude. Muriel Spark's *The Girls of Slender Means* and Anita Brookner's *Don't Look At Me* both share an atmosphere with Taylor's novel, though Spark and Brookner are concerned with youth and Taylor with its opposite. This middle‐class setting, the preoccupation with the lives of women, an attentiveness to the domestic, have led to Taylor's novels being attributed a certain narrowness – ‘I seem to hear the tinkle of teacups’, Saul Bellow is said to have remarked of the novel, when judging the Booker prize – a statement that was seen as a complete condemnation.^14^ ‘By my reckoning, just one cup of tea is drunk in the novel’, writes Michael Hofmann in the introduction to the NYRB edition of *Mrs Palfrey*, ‘and it doesn't tinkle’.^15^ The quality that so offended Bellow may have something to do with gender – Anita Brookner finds in Taylor's writing ‘an unfailing and unmistakable female intelligence’,^16^ Rosamund Lehmann calls it ‘a piercing feminine wit’.^17^ What is this female intelligence? Perhaps it has something to do with Taylor's quality of attention. After all teacups, and all that they signify, are as good a starting point as anything else for a diagnosis of English political life. And indeed, from the very beginning of Taylor's novel, the Claremont Hotel on Cromwell Road is the setting for another scene.

## England's manners

Mrs Palfrey is first introduced to the reader as ‘a tall woman with big bones and a noble face, dark eyebrows and a neatly folded jowl. She would have made a distinguished‐looking man and, sometimes, wearing evening dress, looked like some famous general in drag.’^18^ Her choice of clothes through the novel adds to this impression. We learn of her short fur coat, which always smelt of ‘camphor and animal’ and ‘her evening dress with metallic beads down her sloping breast’.^19^ Taylor's original comparison of Mrs Palfrey wasn't just to any general. Taylor described Mrs Palfrey looking like ‘Lord Louis Mountbatten in drag’.^20^ Taylor's biographer, Nicola Beauman, notes that the novel's publishers asked for this sentence to be changed. Taylor complied, and wrote back saying ‘I didn't know about the gossip (we live very quietly)’.^21^ Even a cursory glance at the outfit Mountbatten wore to his swearing in ceremony as the Viceroy of India confirms the satirical resemblance to the fictional Mrs Palfrey. Known for his love of clothes and imperial bling, Mountbatten is pictured in a fur‐lined cloak over his naval dress, his many medals and braids gleaming across his chest. In a photograph in the National Portrait Gallery, taken in 1937 to mark the coronation of George VI, he is pictured in a similarly improbable quantity of gold, though this time in a cloak in blue and maroon.

The scene of Mrs Palfrey's arrival at the Claremont is intercut with memories of her arrival in Burma as the wife of a colonial official:

She had always known how to behave. Even as a bride, in strange, alarming conditions in Burma, she had been magnificent, calm – when (for instance) she was rowed across floods to her new home; unruffled, finding it more than damp, with a snake wound around the banister to greet her. She had straightened her back and given herself a good talking‐to, as she had this afternoon in the train.^22^

Lord Mountbatten, was of course given the title of Earl Mountbatten of Burma in 1947.

On her first evening at the Claremont, Mrs Palfrey accompanies the residents into the television room, where she sits on a stiff chair behind the more comfortable armchairs. In front of her, ‘(h)eads with thinning hair rested on the antimacassars’.^23^ In her novel *The Return of the Soldier* (1918), Rebecca West had already deployed the antimacassar to summon an atmosphere of a fading, and faded world: ‘a mob of female relatives who were all useless either in the old way, with antimacassars, or in the new way, with golf‐clubs’.^24^ The naval and colonial associations of macassar oil (the hair product made with raw materials from the Dutch East Indies) are not superfluous here. They reinforce the imperial geography of the Burma and Indonesia which lies beneath the South Kensington hotel. Awareness of reduced circumstances plagues the residents of the Claremont. Something has been lost. Stockings splashed by a car, Mrs Post exclaims about ‘England's manners’ – ‘What happened to them? They used to be so good.’ Mrs Palfrey is sympathetic. And the colour of Mrs Post's stockings? – ‘Gunmetal’.^25^

For Mrs Palfrey in particular, the loss of her station is inextricably linked with loss of the British Empire. The colonial scenes in which Mrs Palfrey spent a considerable part of her life haunt her time at the Claremont. Finding Mr Oswald's swearing distasteful (he describes the Claremont's bread and butter pudding as ‘bloody awful’), Mrs Palfrey finds herself thinking of her husband, who had ‘never sworn before her, although she was sure he had often done so, at the right time, in the right place. She vaguely envisaged recalcitrant natives.’^26^ We learn of Mrs Palfrey's indifferent cooking: ‘She had never been a good cook, for in the East it had been done for her.’^27^ The lack of visits from her grandson lead to the deployment of tactics learnt in the colonies: ‘Saving face had been an important part of her life in the Far East, and Mrs Palfrey tried to save hers now.’^28^

The extent of Mrs Palfrey's loss becomes clear when she is writing to her daughter Elizabeth, who lives in Scotland. Mrs Palfrey cannot understand her daughter's enthusiasm for the place, she has ‘surrendered herself’ to Scotland, ‘a strange reaction to a foreign country’.^29^ For her own part, Mrs Palfrey, when she had been abroad, was conscious of her origins: ‘“I am English”. She had kept *that* barrier up’.^30^ She has lost her daughter to Scotland, but in writing to her she becomes aware of another loss: ‘When she was young, it had seemed that nearly all the world was pink on her school atlas – “ours”, in fact. Nearly all ours! she had thought. Pink was the colour, and the word, of well‐being, and of optimism.’^31^ In 1850, the booksellers Fullarton and Co. began to use pink to depict the British Empire in their maps, an innovation that became so popular that it was widely reproduced over the next 100 years. For Mrs Palfrey, the world has changed, quite literally. This loss of the world in pink, of empire, and indeed of her station is life is accompanied by a fundamental loss of the self: ‘When she was young, she had had an image of herself to present to her new husband, whom she admired; then to herself, thirdly to the natives (I am an Englishwoman). Now, no one reflected the image of herself, and it seemed diminished: it had lost two‐thirds of its erstwhile value (no husband, no natives).’^32^

Elizabeth Taylor is inviting us to read the loneliness of the Claremont Hotel in relation to England's colonial history, the ways in which Mrs Palfrey has brought to the Claremont the ghosts of natives past. Her inner dialogue is peopled with the colonial subjects amongst whom she lived, in opposition to whom she defined herself. She is now without the gaze of the colonial other, a gaze that props up at least a third of her being. Though it is Mrs Palfrey's motto in life to ‘not give in to melancholy’, the psychoanalytic writing around melancholia provides a useful way into thinking about Mrs Palfrey's reaction to the loss of empire.

## Cucumber sandwiches that repeated

Freud's essay on ‘Mourning and Melancholia’ (1917) describes mourning as ‘the reaction to the loss of a loved person, or to the loss of some abstraction which has taken the place of one, such as one's country, liberty, an ideal, and so on’.^33^ Freud's account of the experience of loss places it squarely within a social, indeed political field. This is not surprising, given that the essay was written after the outbreak of the First World War, and can be read alongside a series of essays in which Freud considers the psyche in relation to questions about the social that have been posed by the war. The essay makes a distinction between the experience of mourning, and that of melancholia. Mourning, though painful and slow, is finite – and the object of mourning, that is, who or what has been lost, is known. Melancholia, on the other hand, drags on and on, and involves ‘an object loss that is withdrawn from consciousness’.^34^ Further, because the person who has suffered the loss is so mixed up with what has been lost, so identified with it, melancholia is also an experience of loss of the self. For Freud, there is an oral quality to identification:

identification is a preliminary stage of object‐choice, that it is the first way – and one that is expressed in an ambivalent fashion – in which the ego picks out an object. The ego wants to incorporate this object into itself, and, in accordance with the oral or cannibalistic phase of libidinal development in which it is, it wants to do so by devouring it.^35^

Melancholic suffering involves the difficulties of incorporation, the problem of what to do with the effects of the object that has been taken in. Melanie Klein, the psychoanalyst most associated with theories of object relations, places a similar emphasis on incorporation in the experience of depression: ‘both in children and adults suffering from depression, I have discovered the dread of harbouring dying or dead objects (especially the parents) inside one and an identification of the ego with objects in this condition’.^36^ In her book *Black Sun: Depression and Melancholia*, Julia Kristeva describes a ‘cannibalistic solitude’, suggesting that it is possible to swallow an object who prevents any sort of relation with another. The object trapped within makes it impossible for another to get through. The ‘melancholy cannibalistic imagination’, Kristeva writes, ‘is a repudiation of loss's reality’.^37^

Time, so slow to pass in the Claremont, is divided up into mealtimes: ‘food made the breaks in the day’.^38^ The residents make a ritual of checking the menu, and commenting on it to each other. There is, however, something indigestible about food at the Claremont. We're told it is barely better than an English boarding school, and Taylor's descriptions of ‘pasty celery soup’, ‘wobbling red jellies’, ‘slopping fruit salad’ inspire disgust rather than desire.^39^ There is also something emphatically English about the dishes: ‘roast Surrey fowl’, ‘cold Norfolk turkey’, and cucumber sandwiches.^40^ Much as the residents of the Claremont look forward to the food, they also suffer from it. Taylor is precise in her descriptions of the ways in which the food doesn't sit well. Mr Oswald suffers from ‘acid gurglings’, his omelette churning in his stomach.^41^ Mrs Post has to laugh to cover up a fart. And on two occasions in the text, we are told about the Claremont's ‘cucumber sandwiches that repeated’.^42^

In *The Melancholy of Race*, Anne Anlin Cheng suggests that Freud's account of melancholia presents an ‘apt paradigm for elucidating the activity and components of racialization’.^43^ Extending the account of ‘uncomfortable swallowing’ that Freudian melancholia entails, she writes that, in order to ‘sustain the fiction of possession’, the loss of the object must be denied.^44^ And yet, when the object is incorporated, the possibility of its return would ‘jeopardize the cannibalistic project’.^45^ Cheng writes: ‘although it may seem reasonable to imagine that the griever may wish for the return of the loved one, once this digestive process has occurred, the ego may in fact not want or cannot afford such homecoming’.^46^ Thus, incorporation is accompanied by exclusion: ‘(l)ike melancholia, racism is hardly ever a clear rejection of the other. While racism is mostly thought of as a kind of violent rejection, racist institutions in fact often do not want to fully expel the racial other; instead, they wish to maintain that other within existing structures.’^47^ Cheng's account of melancholia is offered as a critique of American national identity, though her suggestion that colonialism makes the issue of racial identity a question of place is relevant to a reading of Taylor's novel, where the Claremont Hotel has been saturated with an imperial geography.

Like Cheng's reading of melancholy, Taylor invites us to read indigestion at the Claremont as a symptom of the body politic. If the hotel itself, and the lost colonies of the ‘Far East’ provide two co‐ordinates with which to map the dynamics of loss and exclusion that Taylor presents, then the case of Mr Osmond, who suffers from acid gurglings, presents a third key dimension. Mr Osmond is wont to complain about the accent in which the weather report is read: ‘I don't want a damned Aussie telling me about my English weather’.^48^ He writes about his discontents to the *Daily Telegraph*, which once printed his letter about ‘the distribution of Fritillaria Meleagris in the South of England’.^49^ His other letters on decimalisation, fluoridation, artificial insemination, the migration of birds, racial integration, drugs, and thuggery (with the interesting derivation of the word ‘thug’) are all ignored by the newspaper. Mr Oswald is preoccupied with what is properly English – accents, etymologies, and even plants – the fritillaria meleagris being the subject of some debate about whether it is a native British plant.

Mr Osmond is concerned with demarcating the authentically English – and the implications of this come into sharper focus when one of his letters (to the *Evening News*) is published: ‘It was about foreigners receiving free medical treatment in England, which he personally was not prepared to subsidise.’^50^ Read in the light of the contemporary vilification of migrant mothers, especially their use of the NHS, Taylor's observations about the insular nationalism of Mr Oswald are particularly sharp and far‐reaching. Following Cheng, we may suggest that Mr Osmond's acid gurglings, the omelette churning in his stomach, have something to do with his inability to accept into his idea of what is English anything that seems ‘other’. The ‘damned Aussie’, the ‘foreigners’ are indigestible in Mr Oswald's English body politic. Another passage in the novel furthers this reading:

Mr Osmond was writing to the *Daily Telegraph*, as usual. ‘We are lucky to have such a fine body’, he wrote, ‘of men’, he added, after taking a sip of wine. Mrs Palfrey, depressed, watched him. Action, she thought. He is taking action, he is expressing himself, keeping himself going. Some indignant thought made him give a little snort.^51^

We are not told why Mr Osmond is concerned with this fine body of men, just as we are not told what was so objectionable about ‘the doctor I was forced to have attend me in Paris’.^52^ Like her use of punctuation to portray solitude and the slow passing of time at the Claremont, Taylor deploys silences to suggest something about Mr Oswald's beliefs. We can wonder if the missing signifier in these sentences is race, but precisely in leaving it missing Taylor captures the allusive, ellipsis driven quality of exclusionary politics in Britain, something that is conveyed through implication – indeed, Mr Osmond is often described nodding and winking at waiters as he draws them aside to tell sexual jokes.

In 1959, Oswald Mosley was a candidate for Member of Parliament for the former constituency of Kensington North, a short distance from the Claremont Hotel. *Action* had been the newspaper of the British Union of Fascists in the 1930s, and was once again the organ of the Union Party. Mosley conducted his campaign on an anti‐migrant platform (‘the Government's policy of permitting unlimited coloured immigration was a grave error which would inevitably cause trouble’), seeing the election as a chance for British people to ‘express their opinion on the acute question of coloured immigration’.^53^ Mosley lost, and got the lowest percentage of the vote in the elections. Though he hints at electoral fraud in his memoirs, he accepted the election result. In his account of the time, published in his memoir *My Life*, he is insistent that his policies were not of ‘racialism’ but addressing unemployment and overcrowded housing. Even in Mosley's writing – and he is hardly shy of his opinions – we can see the elliptical, metonymic relation to race that Taylor captures in the speech of Mr Osmond.

Indeed, depictions of Mr Osmond in the novel bring to mind a kind of Mosleyan politics. There is even a consonance in their names, between Osmond and Oswald. At another point in the novel, describing Mrs Post's poor memory, Taylor draws attention to a similar word game: ‘she got Elizabeth Bowen muddled with Marjorie Bowen, and could never remember that there were two Mannings and two Durrells and a couple of Flemings’.^54^ Many discussions of *Mrs Palfrey* draw attention to Taylor's descriptions of Mrs Burton and how they are likely an allusion to the film star who was the author's namesake. Taylor was very conscious of her shared name (she often received letters intended for the film star, asking her for photographs), but it says something about the way in which she is read that the indexing of Mrs Burton is widely commented on while the connection of Mr Osmond/Oswald Mosley has gone unremarked.

Describing Mrs Palfrey watching Mr Osmond, Taylor brings together depression, action, expression, indignation. The depression, loneliness, and melancholia I've suggested are symptoms of the loss of empire, a melancholic relation, to a lost object, that is constitutive of the self. Action, expression, and indeed indignation can be read as a response to this melancholia – note Mosley's comment that he was giving the British people a chance to ‘express’ themselves.^55^ Through the figure of Mr Oswald, and the allusion to the name of the newspaper of Mosley's party, Taylor reminds us that such action has its roots in fascism. In fact, Taylor had been an active member of the Communist Party in 1936, when the British Union of Fascists marched upon Cable Street and faced popular resistance from a coalition of anti‐fascist groups, many of which were socialist and communist. Taylor's novel notes, and indeed responds to, this resurrection of *Action* (‘Action, she thought, he is taking action’).^56^

We can say that Taylor is interested in the return of the repressed – the cucumber sandwiches that keep repeating. Though Mosley only won a small share of the vote in North Kensington, Taylor recognised that the animating forces behind his politics had deep roots. Her depictions of the residents of the Claremont are attentive both to the difficulties of ageing and to the melancholia and loneliness which are symptomatic of their relation to empire. By taking us into the heart of what Paul Gilroy has called ‘postimperial melancholia’, Taylor is addressing a zone of silence in British cultural life. As Gilroy puts it, since 1945, ‘the life of the nation has been dominated by an inability even to face, never mind actually mourn, the profound change in circumstances and moods that followed the end of the empire and consequent loss of imperial prestige.’^57^ *Mrs Palfrey* can be seen as a reckoning with precisely this silence. The isolation that the novel depicts is not solely an effect of old age and frailty. Rather, we're invited to read it as part of the ‘considerable moral and psychological cost’, as Gilroy puts it, ‘of the repressed and buried knowledge’ of the violence of empire.^58^ In this, once again, Taylor was ahead of her times.

Ahead of her times in many ways, but also *of* her times, depending on who she is read alongside. While she is placed squarely amongst a group on white women writers with whom she maintained correspondences and friendships – Ivy Compton Burnett and Elizabeth Bowen amongst others, this grouping even as it draws attention to key aspects of her work, may also serve to eclipse other important concerns in her writing. There are, after all, other depictions of London loneliness, notably Sam Selvon's *The Lonely Londoners* and George Lamming's *The Emigrants*. The question isn't whether Taylor read Selvon or Lamming, but that read together, the novels can show the way in which Taylor was in conversation with a different set of literary and political preoccupations.

Two stories Elizabeth Taylor wrote in the 1960s are set in Kensington and Chelsea. The protagonist of ‘Tall Boy’ is Jasper Johns, a West Indian migrant living in a bed‐sit on St Luke's Street. Like *Mrs Palfrey*, this too is a portrait of solitude, and St Luke's Street is but a short walk from the Claremont Hotel. The story hinges around Jasper's attempt to mark and celebrate his birthday in a place where no one knows him. He purchases and posts himself a birthday card – and we also learn that he has bought a bright new tie so that his co‐workers can ask him about it, and he in return can announce his birthday. The person whose birthday it is has to buy everyone cakes for tea, and Jasper has been waiting to do this. Though Jasper is alone in the story, and there is difficulty in bearing such aloneness, the quality of his solitude is notably different from that of the residents of the Claremont hotel. Drinking a beer (and not liking it very much), Jasper's thoughts turn to Londoners: ‘(t)here seemed to be inherent in them a wish for self‐punishment he could not understand – a greyness of soul and taste, to match the climate. Perhaps in total depression there was safety. His own depression – of fits and starts – held danger in it, he guessed.’^59^

Let us take the example of food, again. Jasper cooks three meals over the course of the story, and even though they are at best approximations of what he wants – flour rubbed with dripping and fried in bacon fat, ‘as near as he could get to his mother's fried dumplings’ – there is a sense of nourishment there.^60^ British ingredients can be transformed by him, and his sense of sadness or isolation is not immutable, it can turn into something more exciting, there is a sense that he can find pleasure in his dreary surroundings. The story closes with an image of Jasper eating baked beans, ‘spooning them up contentedly’, looking at a photograph of his three sisters which has unexpectedly arrived in the post accompanying a letter from his mother, along with the birthday card he posted to himself.^61^ In that last scene, the former colony and the imperial country are juxtaposed, and the story celebrates this connection.

‘The Devastating Boys’ tells the story of Septimus Smith, ‘Sep’, and Benny Reece, two children from London, and Laura and Harold, a couple who host them in their house in the country. They've found Sep and Benny through a scheme that offers London children the chance to spend the summer in the countryside. Harold sees this as some kind of anti‐racist project, though is himself totally cut off from the bringing up of children. Laura thinks herself completely unequipped for the situation, and indeed the story shows how she has a less than adequate understanding of the children's circumstances. Taylor conveys this with characteristic lightness of touch. Take for example, this scene around a game of Snakes and Ladders, which Sep has lost to Benny:

‘And what do you do if you lose?’ Laura asked, glancing down at the hearth rug. ‘You can't win all the time.’

In a muffled voice, Sep at last said, ‘I don't win any time. They won't let me win any time.’

It's only luck.

No, they don't *let* me win. I just go and lie down and shut my eyes.^62^

If Laura struggles to understand the realities of racism and migration, the children misunderstand her middle‐class, English evasiveness to comic effect. When Laura says that her house‐help ‘comes in to help with the housework’, Benny replies that she ‘must be a very kind old lady’.^63^ And Sep stops Laura from playing ‘God Save the Queen’ on the piano, quoting his mother: ‘God save *me’*. In the end, it is Laura and Harold's relationship which is reinvigorated by the visit from the boys. Though both stories sometimes navigate questions around race and migration in language that may be jarring in the present day, they unreservedly celebrate the figure of the migrant.

## Mrs Palfrey must fall

Is there a way out of Mrs Palfrey's melancholia? Taylor is interested in finding one. The Claremont Hotel, where Mrs Palfrey has experienced such loneliness, becomes the site of intersecting solitudes when she invites Ludovic Myers, a young writer, to play the part of her grandson. Desmond, her grandson, though very much a resident of the city, has not been replying to his grandmother's invitations and has left her exposed to the pity of the other residents. Mrs Palfrey and Ludo find themselves in an unlikely friendship, engaged in a game of deceiving the other residents at the Claremont. Moreover, the young and impoverished Ludo had an appetite, even for the Claremont's food.

In Ludo's company, Mrs Palfrey begins to regain her capacity for pleasure. She buys cheese at Harrods, laughs, surprises herself. She breaks her usual codes of conduct, in taking an extravagant taxi, in lending Ludo money. In many ways, the novel dramatizes and narrates the encounter between the solitude of the young man and the old woman. This friendship however, sets other changes afoot which take us back to Taylor's critique of postcolonial melancholia. On Sunday, the residents of the Claremont sit watching television. This is how Taylor describes the scene:

There was usually a demonstration on Sundays, with milling crowds in Trafalgar Square and forays into Downing Street. The policemen and the horses were always sympathised with. They had the Claremont solidly behind them. ‘Oh, those poor horses!’ Mrs Post kept exclaiming. ‘What have they ever done to deserve this?’.^64^

This time however, Mrs Palfrey's allegiances lie elsewhere: ‘Mrs Palfrey, with her new stake in youth, said nothing’.^65^ Later in the novel, there is another indication of shifting loyalties. Lady Marjorie Swayne has come to stay at the hotel, and taken to Mrs Palfrey. Unlike the residents of the Claremont Lady Swayne is relentlessly social, and lets it be known. When she mentions an editor at *The Sunday Times*, Mrs Palfrey says ‘I take the *Observer*.’ This surprises Lady Swayne, who says ‘I'm afraid we gave that up at the time of Suez.’ Lady Swayne's response draws attention to the newspaper's position over the Suez crisis, and their exposé of British falsehoods during the Second Arab‐Israeli war. The choice of the *Observer* has another connection with Mrs Palfrey's past. George Orwell was a frequent contributor to the newspaper.

Orwell served as police office in Burma, and was a well‐known critic of British imperialism. Mrs Palfrey's invocations of ‘natives’ are in stark contrast to what Orwell had to say on the subject. Describing the place of the British officer in the colony, he writes: ‘it is the condition of his rule that he shall spend his life in trying to impress the ‘natives’, and so in every crisis he has got to do what the ‘natives’ expect of him. He wears a mask, and his face grows to fit it.’^66^ Burma, for Orwell, was a place where he saw ‘the dirty work of Empire’.^67^ In what seems like a rare intervention of authorial judgement – or a sea change in Mrs Palfrey's opinions, Taylor calls Lady Swayne's opinions ‘bigoted’ and ‘self‐congratulatory’.^68^

The narrative of Mrs Palfrey at the Claremont is organised around two falls. The first, while Mrs Palfrey is out fetching a novel for Mrs Arbuthnot, introduces her to the young Ludovic Myers, who rescues her. The second fall comes towards the end of the novel, and is the beginning of the end. Remember that Mrs Palfrey has been introduced to the reader as ‘a famous general in drag’. This is how Mrs Post remembers her fall:

(t)o see Mrs Palfrey, of all people, being carried down those public steps, in broad daylight, across the common pavement, with people staring (as Mrs Palfrey in her great pain, feeling blood creeping down from her brow again, had realised they would) – that had upset them all. It was like watching a famous statue topple over. Prone, and broken, she was hardly Mrs Palfrey.^69^

By this time something has changed in Mrs Palfrey, and to some extent, in the Claremont, there is a greater sense of connection amongst the residents. Yet Taylor's observations about post‐imperial melancholia suggests that Mrs Palfrey *must* fall, for her own sake, and so that the colonial investments of present‐day Britain may also begin to crumble.

A few pages later, we are given another image of Mrs Palfrey. Ludo, picturing Mrs Palfrey's fall based on the accounts he has heard at the Claremont, thinks of the Soviet film *Battleship Potemkin*, where an old woman at a protest demonstration is shot by cavalry. In her final days, and the final pages of the novel, Mrs Palfrey is freed from the colonial associations that have accompanied her through the novel. She uses her capital (‘never touch capital’) to pay for a comfortable hospital room, refuses a visit from Mr Osmond, and her thoughts are about Ludo, not her husband, the natives, or Burma. Ludo, for this part, returns the £50 that he has borrowed from Mrs Palfrey. She considers leaving money for him, but dies before that can be accomplished. There are some things, Elizabeth Taylor suggests, that are best not inherited.

## Notes

1 Nicola Beauman, *The Other Elizabeth Taylor* (London: Persephone Books, 2009), 70.

2 Elizabeth Taylor, *At Mrs Lippincote’s* (London: Virago, 2006), 119.

3 Elizabeth Taylor, *Mrs Palfrey at the Claremont* (London: Virago, 2012), 2.

4 Ibid., 8.

5 Ibid., 5.

6 John Wiltshire, *The Hidden Jane Austen* (Cambridge: Cambridge University Press, 2014), 9.

7 Taylor, *Claremont*, 23.

8 Ibid., 19.

9 Ibid., 20.

10 Ibid., 19.

11 Ibid., 20.

12 Ibid., 44.

13 Ibid., 73.

14 Beauman, *Other Elizabeth Taylor*, 369.

15 Michael Hofmann, ‘No Country for Old Women’, 2021. https://thebaffler.com/latest/no‐country‐for‐old‐women‐hofmann.

16 Anita Brookner, ‘Shy of the Crowd’, *The Spectator*, 2012. https://www.spectator.co.uk/article/shy‐of‐the‐crowd/.

17 Beauman, *Other Elizabeth Taylor*, 171.

18 Taylor, *Claremont*, 2.

19 Ibid., 172.

20 Beauman, *Other Elizabeth Taylor*, 363.

21 Ibid.

22 Taylor, *Claremont*, 2.

23 Ibid., 7.

24 Rebecca West, *The Return of the Soldier* (Mineola, NY: Dover, 1918).

25 Taylor, *Claremont*, 9.

26 Ibid., 13.

27 Ibid., 152.

28 Ibid., 18.

29 Ibid., 104.

30 Ibid.

31 Ibid.

32 Taylor, *Claremont*, 3.

33 Sigmund Freud, ‘Mourning and Melancholia’, in *The Standard Edition of the Complete Works of Sigmund Freud*, vol. 14 (London: Hogarth Press, 1955), 243.

34 Ibid., 245.

35 Ibid., 249–50.

36 Melanie Klein, ‘A Contribution to the Psychogenesis of Manic Depressive States’, in *Love Guilt and Reparation and Other Works 1925–1941* (New York: Macmillan, 1975), 266.

37 Julia Kristeva, *Black Sun: Depression and Melancholia* (New York: Columbia University Press, 1992), 12.

38 Taylor, *Claremont*, 12.

39 Ibid., 5.

40 Ibid.

41 Ibid., 132.

42 Ibid., 51, 109.

43 Anne Anlin Cheng, *The Melancholy of Race: Psychoanalysis, Assimilation, and Hidden Grief* (Oxford: Oxford University Press, 2000), 10.

44 Ibid., 9.

45 Ibid.

46 Ibid.

47 Ibid., 12.

48 Taylor, *Claremont*, 51.

49 Ibid., 122.

50 Ibid., 78.

51 Ibid., 67.

52 Ibid., 79.

53 Oswald Mosley, *My Life* (London: Nelson, 1968).

54 Taylor, *Claremont*, 23.

55 I am indebted to Helen Tyson’s discussion of ‘action’ in ‘“Action […] Real and Effective”: Marion Milner and “Expressive Action”’, *Critical Quarterly*, 63:4 (2021), 20–32.

56 Taylor*, Claremont*, 67.

57 Paul Gilroy, *Postcolonial Melancholia* (New York: Columbia University Press, 2005), 90.

58 Ibid., 94.

59 Elizabeth Taylor, ‘Tall Boy’, in *Complete Short Stories* (London: Virago, 2012), 495.

60 Ibid., 496.

61 Ibid., 499.

62 Elizabeth Taylor, ‘The Devastating Boys’, in *Complete Short Stories* (London: Virago, 2012), 463.

63 Ibid., 464.

64 Taylor, *Claremont*, 52.

65 Ibid.

66 George Orwell, ‘Shooting an Elephant’, https://www.orwellfoundation.com/the‐orwell‐foundation/orwell/essays‐and‐other‐works/shooting‐an‐elephant/.

67 Ibid.

68 Taylor, *Claremont*, 81.

69 Ibid., 195.

